# Philippine Performance Evaluation and Assessment Scheme (PPEAS): Experiences in Newborn Screening System Quality Improvement

**DOI:** 10.3390/ijns6040095

**Published:** 2020-12-11

**Authors:** Carmencita D. Padilla, Bradford L. Therrell, Karen Asuncion R. Panol, Riza Concordia N. Suarez, Ma. Elouisa L. Reyes, Charity M. Jomento, Ebner Bon G. Maceda, Jovy Ann C. Lising, Frederick David E. Beltran, Lita L. Orbillo

**Affiliations:** 1 Newborn Screening Reference Center, University of the Philippines Manila, Metro Manila 1000, Philippines; krpanol@up.edu.ph (K.A.R.P.); rnsuarez@up.edu.ph (R.C.N.S.); mlreyes9@up.edu.ph (M.E.L.R.); cmjomento@up.edu.ph (C.M.J.); egmaceda@up.edu.ph (E.B.G.M.); jmcasamorin@up.edu.ph (J.A.C.L.); fbeltran@up.edu.ph (F.D.E.B.); 2 Department of Pediatrics, College of Medicine, University of the Philippines Manila, Metro Manila 1000, Philippines; 3 Department of Pediatrics, Medical School, University of Texas Health Science Center at San Antonio, San Antonio, TX 78229, USA; therrell@uthscsa.edu; 4 National Newborn Screening and Global Resource Center, Austin, TX 78759, USA; 5 Department of Health, Metro Manila 1003, Philippines; litaorbillo@gmail.com

**Keywords:** newborn screening, performance evaluation, quality improvement, Philippines

## Abstract

Newborn Bloodspot Screening (NBS) has existed for over 60 years, having been initiated by Guthrie in the U.S. In the Philippines, NBS was introduced in 1996 and later was supported by legislation. The NBS program now includes 29 conditions, covering 91.6% of the newborn population in 2019. Program growth and expansion necessitated development of a formal performance evaluation and assessment scheme (PEAS) for monitoring performance and for continuously improving quality. This study’s objective was to present the development, implementation, and results to date of the Philippine Performance PEAS (PPEAS). Using the comprehensive listing of laboratory and non-laboratory elements in the model PEAS system in the U.S., PPEAS tools were developed for critical Philippine NBS system components: regional Department of Health (national health agency, Philippines) (DOH) offices (CHDs), NBS laboratories (NSCs), NBS specimen submitters (NSFs), and long-term case management centers (NBSCCs). Data generated from the various PPEAS have been periodically reviewed and analyzed for NBS system impact. PPEAS were developed to facilitate quality improvement at various levels of the Philippine NBS system. PPEAS identified successes, gaps, and challenges to be addressed by NSCs, NSFs, CHDs, and NBSCCs with the assistance of the Newborn Screening Reference Center and the Department of Health.

## 1. Introduction

**Figure 1:**
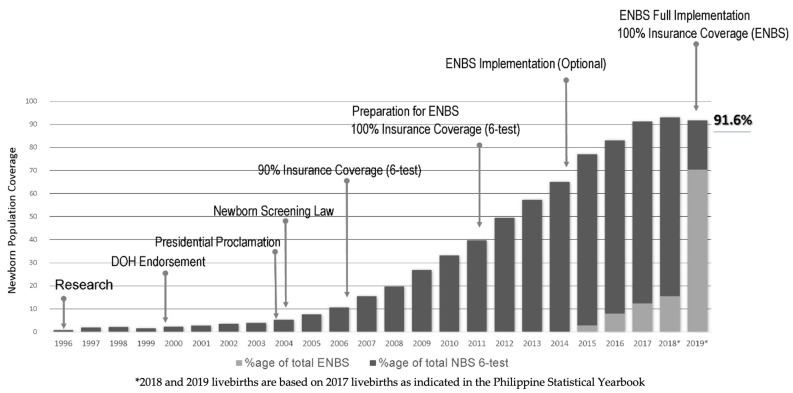
Annual trend in Philippine Newborn Bloodspot Screening (NBS) coverage and program milestones.

Newborn Bloodspot Screening (NBS) has existed for over 60 years, having first been initiated by Guthrie in the U.S. [1,2]. In the intervening years, sustainable NBS has spread widely in countries with developed economies, but its implementation in the developing world has been slow [3]. The history of NBS in the Philippines is relatively short (Figure 1). NBS began as a research pilot in 1996, received Department of Health (DOH) approval and support in 1999, Presidential support by presidential proclamation in 2003, and enactment of a NBS law requiring offering of NBS in 2004, the Newborn Screening Act of 2004 (Republic Act No 9288) [4,5,6,7]. Initially funded through a fee, inclusion as a newborn health benefit by national health insurance (PhilHealth) in 2006 provided the impetus for increases in population coverage for the basic 5-test screening panel (phenylketonuria (PKU), glucose-6-phosphate dehydrogenase (G6PD) deficiency, congenital hypothyroidism (CH), congenital adrenal hyperplasia (CAH), and galactosemia (GAL)) [8]. Maple syrup urine disease (MSUD) was added to the screening panel in 2012 [9] and an Administrative Order from DOH provided guidelines for implementing expanded NBS (ENBS) for a total of 28 conditions in 2014 and further to 29 conditions in 2018 [10,11]. While the initial cost of expanded screening beyond the base panel required payment of a fee, inclusion of the fully expanded panel as a national insurance benefit began in 2019 [12]. As a result, uptake of the expanded screening panel has risen dramatically with newborn population coverage currently exceeding 90% (Figure 1).

NBS is generally accepted as a smoothly integrated 6-part system that includes education (parents, healthcare providers, policy makers), screening (including specimen collection, transport, and laboratory analysis), short-term follow-up (tracking and further testing when necessary), diagnosis (including medical specialists), management (treatment and/or other medical care), and evaluation (long-term follow-up/outcomes and systems review) [13]. A model comprehensive performance evaluation and assessment scheme (PEAS) also exists as a guide for NBS system improvement [14]. While the initial Philippine NBS system developed in an academic environment, its migration to, and inclusion in, the public health system was essential for its long-term sustainability and expansion at the national level [7].

**Figure 2:**
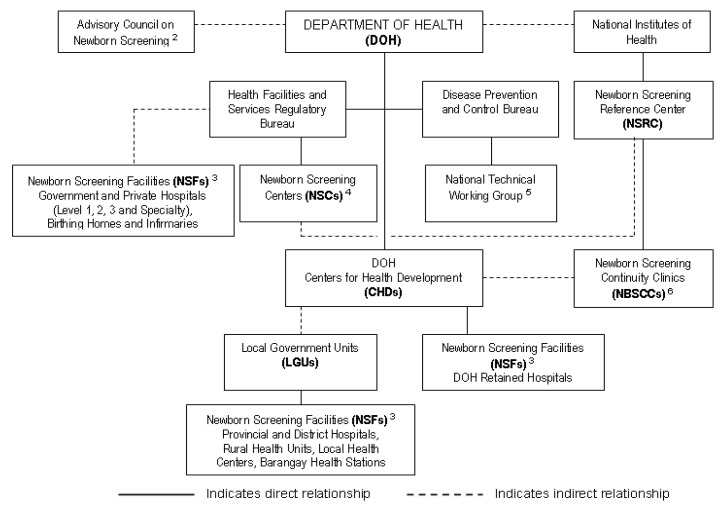
National Comprehensive Newborn Screening System ^1^ (NCNBSS) Functional Chart.
^1^ “NCNBSS shall ensure that every baby born in the Philippines is offered the opportunity to undergo newborn screening and thus be spared from heritable conditions that can lead to mental retardation and death if undetected and untreated” [17,18].^2^ According to the Newborn Screening Law, the Advisory Committee on Newborn Screening was, “… created and made an integral part of the Office of the Secretary of the Department of Health (national health agency, Philippines) (DOH) to ensure sustained inter-agency collaboration. The committee reviews annually and recommends conditions to be included in the newborn screening panel of disorders; reviews and recommends the newborn screening fee to be charged by Newborn Screening Centers; reviews the report of the Newborn Screening Reference Center on the quality assurance of the Newborn Screening Centers and recommends corrective measures as deemed necessary. The Committee shall be composed of eight (8), including the Secretary of Health, who shall act as the chairperson. The other members of the of the Committee shall be as follows: (1) the Executive Director of the National Institutes of Health (NIH) who shall act as a Vice Chairperson, (ii), an Undersecretary of the DILG (Department of the Interior and Local Government); (iii) the Executive Director of the Council for the Welfare of Children; (iv) the Director of the Newborn Screening Reference Center; (v) three (3) representatives appointed by the Secretary of Health who shall be a pediatrician, obstetrician, endocrinologist, family physician, nurse or midwife from either a public or private sector. The three (3) representatives shall be appointed for a term of three (3) years, subject to their being reappointed for additional three (3) year periods for each extension” [15].^3^ A Newborn Screening Facility (NSF) is a health facility that educates parents about NBS during prenatal visits, collects blood specimens for NBS, sends specimens to the Newborn Screening Center (NSC), and recalls patients found positive for NBS and assists in managing patients.^4^ A Newborn Screening Center (NSC) is a facility equipped with a NBS laboratory that complies with standards established by NIH and DOH and provides required laboratory tests and recall/follow-up for newborns with conditions identified by NBS [17].^5^ The National Technical Working Group on Newborn Screening has the goal of the long- and medium-term target setting and planning of the National Comprehensive NBS System. It ensures that all policies and standards of the program adhere to overall internationally accepted standards and ethical considerations [19,20,21].^6^ Newborn Screening Continuity Clinics (long-term follow-up) (NBSCCs) are administratively under the NBS Reference Center (NSRC) with referrals from the NSCs. DOH offices (CHDs) assist the NBSCCs in monitoring patients. NBSCCs are located in government hospitals administratively under the CHDs [18].

The Philippines is an archipelago of over 7600 islands divided into three main groups from north to south (Luzon, Visayas, Mindanao), and 17 governmental administrative divisions resulting in 17 public health regions. There are approximately two million births annually. The Philippine NBS system includes a formal NBS program at the Department of Health (DOH), which provides policy development, program direction and quality oversight [15]. NBS laboratory accreditation is also a responsibility of the DOH, and external laboratory proficiency is provided by the U.S. Centers for Disease Control and Prevention (CDC) and Taiwan’s Preventive Medicine Foundation (TPMF: external quality assurance for NBS quantitative G6PD). The multi-component National Comprehensive NBS System (see Figure 2) currently includes: (1) a NBS Reference Center (NSRC) that provides national program management, program review, and technical assistance; (2) seven NBS Centers (NSCs) that collaborate to provide laboratory testing; (3) 17 DOH Regional Offices or Centers for Health Development (CHDs) that provide local and regional assistance in education, local and regional implementation, and both short- and long-term follow-up; (4) over 7000 NBS Facilities (NSFs) that provide prenatal education, specimen collection, and assistance with short-term follow-up and case management; and, (5) 14 NBS Continuity Clinics (NBSCCs) that monitor and provide long-term follow-up and case management assistance, including testing support for indigents [16].

Implementation of the NBS system has included extensive planning, broad-based education of professionals, the public, and policy makers, employment of dedicated public servants, and development of laboratory, follow-up, and communications capabilities, which includes carefully thought out program and public health policies. Public and parent input into system development are welcome and primarily occur through the at-large appointees of the DOH Secretary to the NBS Advisory Committee. A representative of the Philippine Society for Orphan Disorders routinely participates in the activities of the National Technical Working Group on Newborn Screening.

**Figure 3:**
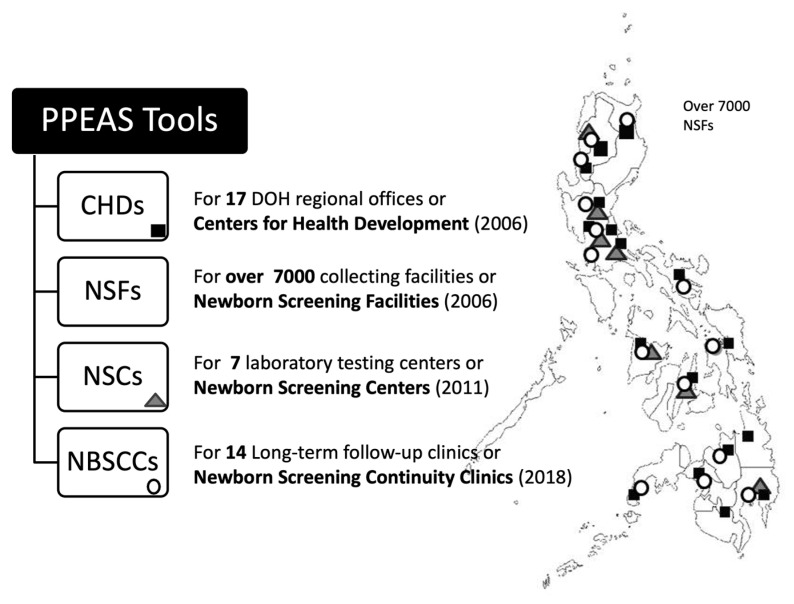
Identification on the various entities involved in Philippine NBS and Philippines performance evaluation and assessment scheme (PPEAS) evaluations.

In order to provide a means of goal setting, performance monitoring and quality improvement, the NSRC sought out models of mechanisms that might accomplish a comprehensive performance evaluation and assessment activity from more developed NBS programs around the world. In particular, the U.S. Evaluation and Assessment Scheme (PEAS) [14], developed as a federally funded initiative, provided a model comprehensive tool on which to base a similar activity in the Philippines more targeted to local program elements and needs. Utilization of PEAS as a template led to the development of a multi-faceted Philippine PEAS (PPEAS). Development of PPEAS evaluation tools (Figure 3) has resulted in periodic assessments of each program facet as a means of evaluating the various components of the Philippine NBS system, identifying gaps in service provision, and developing and initiating quality improvement [22]. In this article, we review the development of PPEAS, its successful implementation in the Philippines, and examples of findings that have resulted in NBS system quality improvement.

## 2. Methodology

Development of PPEAS began in 2005 as a collaboration between the Philippine NSRC and the U.S. National Newborn Screening and Global Resource Center (NNSGRC) [22]. Beginning with a multi-session retreat/workshop involving both Centers, insight into preparation of the U.S. developed PEAS [14] was provided and a detailed outline of the Philippine NBS system was discussed to identify similarities and differences. PEAS includes a comprehensive itemized listing of NBS system components developed by multi-disciplinary working groups focused on laboratory and non-laboratory considerations in a well-developed NBS system. It is divided logistically into pre-analytical, analytical, and post-analytical elements, and includes a section of cross-cutting considerations (i.e., personnel, education, etc.). Additionally, whether or not an itemized component can be monitored or evaluated quantitatively is indicated. Understanding and assessing each component of the U.S. PEAS not only provided a basis for developing quality improvement tools for the Philippine NBS program, but also provided a more in-depth understanding of the complexities of some NBS system components that may have been previously overlooked or not fully understood. Drawing from various U.S. PEAS sections, a PPEAS was created by the NSRC, with considerable input from both DOH and NSC personnel, to serve as a tool(s) to assist in goal setting and better defining and evaluating the quality of critical Philippine NBS system components.

Both PEAS and PPEAS define various program needs in detail using a format that defines major program components and each of the elements necessary to successfully assess the degree of implementation and ongoing maintenance of that component [14,22]. Specific PEAS components associated with various Philippine NBS elements were extracted, modified if necessary, and inserted into various sections of PPEAS (see summary of major PPEAS considerations in Table 1). Each section was aimed at detailing responsibilities of the various operational units involved in implementing and sustaining NBS screening in the Philippines and providing an appropriate way to evaluate and improve NBS, including goal setting where appropriate. Initially, in 2006, PPEAS sections and their evaluation tools were designed to provide guidance and performance monitoring tools for CHDs (regional DOH offices) [23], NSCs (screening laboratories) [24], and NSFs (specimen submitters) [25] aimed primarily at increasing population coverage, improving specimen quality, and ensuring adequate follow-up. PPEAS tools have been modified over time to better meet program needs and to respond to other outside factors such as travel restrictions.

The initial PPEAS evaluation tool for the NSCs emphasized not only laboratory processes (specimen transport, turnaround time, which includes receipt, analysis, results), analytical quality (including positive predictive value), and reporting/patient tracking, but also administrative and finance details, facility operations, record keeping, employee competence, continuing education, and contingency planning [24]. Because CHDs provide coordination between NSCs and NSFs to facilitate and improve screening coverage, timing, specimen quality, and follow-up, their PPEAS tool focused on these areas, including facilitating and improving communications between CHDs, NSRC, NSCs, local governments, and other stakeholders [23]. The large number of NSFs (currently exceeding 7000) presented a special challenge, but their critical role in patient access, specimen collection, and parent education/communication led to the development of a PPEAS tool aimed at improving and harmonizing these pre-analytical and, in some cases, post-analytical activities [25].

The NSRC, with primary system oversight responsibility, in collaboration with the DOH, developed review teams and/or procedures for PPEAS implementation and periodic evaluation monitoring at the national level. For CHDs, evaluation teams included personnel from the NSRC and DOH, with a representative from another CHD and the local NSC included for historic perspective and other pertinent resource information. For NSC evaluation, the review team included NSRC and DOH representatives, with a member of NSC management from a different NSC included to assess technical capabilities. Because NSC screening laboratories must be certified by the DOH, the NSC PPEAS tool also serves for self-evaluation, with the NSRC reviews used for preliminary assessment prior to a formal DOH review. Use of PPEAS to improve quality services of NSFs has been somewhat problematic due to the large number of facilities involved. While initially envisioned as an ongoing CHD quality improvement role in which monitoring/educational PPEAS visits to all of the submitting facilities in a particular region would be accomplished periodically over time, budgetary and travel restrictions have resulted in a modified implementation. Although the goal continues to be periodically evaluating all NSFs, prioritization for PPEAS evaluation is generally given to facilities meeting one of more of the following criteria: large number of unscreened newborns, high unsatisfactory specimen rate, high number of deliveries, new facility, complaints received or investigations ongoing, and/or other issues of special concern (staff training, kit procurement, etc.).

Reviews of CHDs and NSCs are performed according to a predetermined schedule. In each review, the unit under review is given sufficient time to assess its work quality using the appropriate PPEAS tool for self-assessment, including consideration of possible improvement strategies, prior to a review. At the time of a review, the review team verifies the responses to the PPEAS tool and performs an on-sight assessment that includes a formal presentation of pertinent information by the reviewed unit, a physical review of any facility involved, a review of pertinent records, and staff interviews as appropriate. A site visit to a NSF may be performed to validate the monitoring process of the CHD. All findings are discussed within the review team and a consensus report is developed. The review visit concludes with a final summary of findings with management staff that includes a discussion of strengths, weaknesses, and future actions. Review findings are also presented to the CHD Regional Director or NSC Hospital Director as appropriate. A timely formal letter/report to the unit under review follows, and a formal response is required, accompanied by an improvement plan (if appropriate) with dates for milestone accomplishments. The improvement milestones are monitored by the NSRC to ensure compliance with the quality improvement plan. Failure to comply with the assessment scheme can result in punitive actions as necessary.

With the creation and implementation of NBSCCs in 2014, the success of PPEAS for improving the work quality of the three primary screening partners (CHDs, NSCs, and NSFs) led to development of an appropriate NBSCC PPEAS tool in 2018 [26], focused on developing and maintaining quality long-term follow-up functions. In particular, NBSCC PPEAS quality indicators focused on decreasing the number of patients lost to follow-up long-term, ensuring indigent care and services, maintaining meaningful records, and facilitating appropriate inter-program communications (since patients are endorsed from NSCs). Periodic PPEAS reviews are conducted by the NSRC in a manner similar to those conducted for CHDs and NSCs.

## 3. Results

While laboratory quality is relatively easy to evaluate, and the Philippine NBS system has included a well-defined laboratory quality assurance plan along with external international proficiency testing through the CDC and TPMF, and laboratory monitoring/accreditation by the DOH since its beginning, PPEAS provides a way of assessing, documenting, and improving quality not only in the screening laboratory, but also within various non-laboratory components of the screening system.

In 2005, when PPEAS were being developed, only about 7.6% of all newborns were screened for a 5-condition screening panel. Today, at least in part due to the heavy emphasis on improved education, outreach, and quality across the system, over 90% of newborns are screened for the 29-condition panel consisting of endocrine disorders, amino acid disorders, organic acid disorders, fatty acid oxidation disorders, urea cycle defects, thalassemias and hemoglobinopathies, biotinidase deficiency, GAL, G6PD deficiency, and cystic fibrosis [11]. While baseline data were not available for many of the PPEAS elements now routinely analyzed, their recognition as quantifiable quality indicators was critical to program improvement. For example, it was recognized that specimen quality was a major issue in providing timely screening and follow-up. By establishing and monitoring PPEAS indicators assessing the quality of the specimen submitted, including the timeliness of specimen collection, transmittal, and receipt in the screening laboratory, for all entities involved in specimen collection and analysis, the rate of unsatisfactory specimens is now approaching the goal of <1% nationally. Similarly, recognition that specimen and accompanying data were two separate sources affecting the suitability of the specimen resulted in increased emphasis on each element by the entity with direct responsibility for its quality. Additionally, parent, policy maker, and professional education responsibilities and their quality monitoring have resulted in improved understanding of the value of NBS across all groups with the resultant improvement in newborn population coverage.

From the outset, evaluation of the various NBS units has allowed for the identification of issues and challenges met by the NBS program. The CHDs are primarily in charge of conducting NSF monitoring to identify gaps and solutions in the implementation of newborn screening at the level of the health facility. From 2007–2015, annual reviews of CHD performance were performed using PPEAS and onsite reviews. CHDs mainly target NSFs with high unsatisfactory samples and inactive status for monitoring visits. The number of facilities visited is based on lists provided by the NSCs. With the onset of a DOH travel moratorium in 2015, annual reviews included a desk performance review based on data from NSCs, CHD accomplishment reports, work and financial plans, and fund utilization reports. In order to continue responsiveness to PPEAS requirements, CHDs have been active in program implementation reviews (PIRs) and zonal meetings, including a total of 42 such meetings from 2015–2018 across 8 regions. The local NSC is invited to participate. In these meetings, the CHDs are able to review and present data collected as part of PPEAS in order to facilitate interactive discussions aimed at improving screening coverage, specimen quality, and other issues. Additionally, this format provides not only for recognition of problems, but also for recognition of best practices suitable for implementation by others. CHDs also have been able to collaborate internally with other DOH maternal and child health programs and family health cluster meetings. A brief listing of some of the issues and concerns discussed by CHD with the NSFs are included in Table 2.

Utilization of PPEAS for NSCs has perhaps been the most comprehensive, with the tool providing an excellent way in which to gauge potential issues that might arise as part of the DOH formal accreditation process. Heads of the various NSCs have periodically reviewed and updated the individual components of the PPEAS tool and it has served not only for self-evaluation, but also for comprehensive training and education relative to the potentials for quality improvement of NSC operations. Similarly, the PPEAS tool more recently developed for the NBSCCs is serving as an educational tool for the new personnel employed to establish and maintain its operation. Implementation of this new PPEAS has also been useful in NSRC oversight relative to data elements appropriate for performance monitoring. While there are currently insufficient data to fully evaluate the impact of PPEAS on the NBSCC operations, there are increasing indications that as the number of patients endorsed to NBSCCs increases, the number of patients lost to follow-up decreases. It is essential that these types of data be continually collected and analyzed as the program moves forward.

Table 3 provides a selective summary of some of the issues and solutions uncovered across the Philippine NBS system through PPEAS utilization.

## 4. Discussion

Since the development and implementation of PPEAS in 2006, 10 years after the initiation of NBS as a pilot project, considerable progress in NBS systems development has occurred and continues to occur throughout the Philippines. Significant quality improvements are directly attributable to the careful planning and initiation of multiple PPEAS tools. While each tool is designed to meet unique programmatic goals for a specific NBS system partner using detailed performance evaluation and assessment activities, the combined effect of PPEAS has been higher quality, efficient, and effective full population NBS. To this end, the Philippine NBS system has grown from its initial 5-test, fee-based pilot reaching a few hundred newborns in 24 hospitals in Metro Manila to a full population, national insurance covered, 29-test DOH program (established by law), currently reaching over 90% of all Philippine newborns (Figure 1). Readers should note that the apparent decreased program coverage between 2018 and 2019 shown in Figure 1 is an artifact directly attributable to budgetary and payment issues related to the national insurance coverage transitioning from payment for a 5-test panel to payment for a 29-test panel, which resulted in an increased number of patients unable to obtain screening due to costing issues. Data collected thus far in 2020 show a continued return to increased newborn population coverage.

Going forward, PPEAS tools will continue to serve as both an educational and implementation tool aimed at improving the quality of NBS in the Philippines. Improved data collection systems are being implemented that will better focus on collecting harmonized data from all concerned stakeholders taking advantage of the quantitative elements in PPEAS. We have found PPEAS to be effective in evaluating both laboratory and non-laboratory elements within the NBS system. While the U.S. PEAS offers a comprehensive “gold standard” against which to compare performance, quality and complexity of the NBS system, developing programs are cautioned to consider extracting only those elements that can be directly applied in their setting and using the remainder as an aid to future quality growth and development.

## 5. Conclusions

PPEAS was developed to facilitate performance monitoring of quality improvement at various levels of program responsibility. PPEAS has successfully identified a number of program successes as well as gaps and challenges to be addressed by NSCs, NSFs, CHDs, and NBSCCs with the assistance of the NSRC and the Department of Health. PPEAS is continually being used and evaluated for new elements that need consideration and for older elements that may no longer be needed. A comprehensive review of PPEAS data for each affected NBS unit is now considered an important and necessary quality improvement activity to be completed at least every three years as the NBS system continues to grow and improve.

## Figures and Tables

**Table 1:** Summation of the requirements of various Philippines performance evaluation and assessment schemes (PPEAS) by unit.

PPEAS Requirements for CHDs (DOH Regional Offices)	PPEAS Requirements for NSFs (Specimen Collection Facilities)	PPEAS Requirements for NSCs (Laboratory Testing Facilities)	PPEAS Requirements for NSBCCs (Long-Term Follow-up Clinics)
1. Evidence of a usable and appropriate operational structure 2. Enactment of an appropriate plan of action (including financing) 3. Availability of defined systems to support Newborn Bloodspot Screening (NBS) 4. Utilization of a quality health promotion plan for NBS 5. Creation/use of innovative strategies leading to best practices	1. Definition and use of an effective NBS team 2. Creation/use of an organized NBS program within the health facility 3. Specific administrative support for NBS implementation/ continuation 4. Ongoing monitoring and evaluation program for NBS 5. Utilization of a failsafe reporting system to patients and a summary reporting system to NBS Reference Center (NSRC)	1. Adequate and timely service delivery (laboratory and short-term follow-up) 2. A defined education and regulation program 3. Appropriately educated and trained personnel including ongoing education 4. Compliance and proficiency with technical standards 5. Efficient program administration and financing 6. Appropriate and efficient specimen management system 7. Defined useful program linkages 8. Supportive and efficient facility management system	1. A defined multi-disciplinary operational structure 2. Adequate facility support (housing, finances, etc.) 3. Efficient data management system 4. A defined clinical management and referral network 5. NBS program advocacy activities 6. Long-term follow-up data monitoring and evaluation 7. A supportive and efficient administrative system 8. Creation of innovative strategies leading to best practices

**Table 2:** Major issues and concerns arising at regional CHD meetings with NSFs.

Major Issues and Concerns	Recommendations/Agreements
Training	Prioritize NSFs without/limited trained personnel on NBS for NBS trainings Continue improvement of standardized trainings across all NSFs using the standard facilitators guide provided by NSRC Provide/distribute program operations manual
Regional NBS teamwork	Assign appropriate/sufficient NBS staff members at both the CHD/ NSFs Hold regular CHD/NSF meetings to discuss NBS Encourage attendance of NBS team members at national NBS conventions/workshops
NBS coverage	CHDs/NSFs should lobby local government units (LGUs) for increased funding for indigents Increase attention paid to targets set per NSF per CHD region
Unsatisfactory specimens	Conduct more frequent refresher trainings/lectures on proper specimen collection Conduct regular monitoring visits for provincial or city NBS coordinators Reiterate the need for timely collection and transmittal of specimens (avoid batching)
Quality of result transmittal	Clarify need for NSF to relay missing information to NSC by phone followed by letter Clarify when results (normal, abnormal) are released by NSCs to NSFs and who is in charge of releasing results to parents
Advocacy	Strengthen advocacy at NSFs with low uptake of expanded NBS (ENBS) Network and collaborate with other hospital departments regarding NBS CHDs/ NSFs should conduct NBS week celebration and intensify tri media campaigns
ENBS Awareness	CHDs/NSFs should work to increase awareness about ENBS availability and disorders
Confirmatory testing	Remind all stakeholders of the need for immediate patient recall for confirmatory tests Improve follow-up of babies referred to other facilities to assure confirmatory testing Identify glucose-6-phosphate dehydrogenase (G6PD) confirmatory centers in the region
Follow-up	Review and improve monitoring of diagnosed cases
Monitoring	Clarify submission time requirements and indicators needed for quarterly reports Review targets/agreements every 6 months to assess degree of completion
Others/service delivery	Work with NSFs in Geographically Isolated and Disadvantaged Areas (GIDA) to accommodate costs for NBS testing Discuss any concerns regarding conflicts with facility management regarding NBS Develop contingency measures for earthquakes, typhoons, etc., and in case of resignation of staff Address recognition of inactive NSFs (no specimens in previous six months)

**Table 3:** Partial listing of challenges for NSRC identified through PPEAS and the resulting actions taken for quality improvement.

NBS System Challenges Identified Using PPEAS	Corrective Actions Taken and Impact
Variability in level of support across CHDs	Clarified financial policies resulting in greater operational uniformity and expanded support leading to improved program quality → Better specimens and fewer patients lost to follow-up, better turnaround time from collection, send out to the laboratory and recall of patient
Low newborn population coverage in general	Initiated goal setting and standardized training for program personnel with incentives (awards) for meeting or exceeding goals → Participating NSFs and number of newborns served increasing (currently over 7000 NSFs and greater than 90% coverage (most with ENBS)
Low newborn population coverage for expanded ENBS (29 conditions) following its implementation	Identified patient finances identified as inadequate → Able to convince national health insurance to shift coverage from 6-test panel to ENBS 29-test panel (note increase in Figure 1)
Inadequate information on which NSFs are active and their location	Developed and created the NSF online database of information on all facilities offering newborn screening
NSCs lack of uniformity in laboratory testing protocols	Initiated a laboratory standardization effort for laboratory, follow-up, and quality manuals at all NSCs and required development of training plans for the various NSC positions
NSFs lack of uniformity in providing NBS information and services	Created a “Facilitator’s Guidebook” (periodically updated) to aid in standardizing education and service provision among NSFs
Poor specimen quality recognized across many NSFs	Implemented reporting templates for standardizing reports of specimen quality from NSCs and an alert system from NSC to CHD for action in poorer performing NSFs
Uneven work performance across NSCs	Developed a Standard Competency Tool per position/staff to be used in all NSCs
Low recall rate of patients under long-term care	Established Newborn screening continuity clinics (long-term follow-up) NBSCCs in 14 regions in the country resulting in significant improvement in recall rate (from ~30% to >70% recall rate)
	Improved collaboration with pediatric endocrinologists in the referral and management of endocrine patients, and in securing the anthropometric data of their private patients
	Solidified collaboration with the metabolic geneticists for the acute care management of diagnosed metabolic cases
Low treatment compliance among indigent patients	Revised guidelines for the use of CHD fee funds to include other laboratory tests, long-term management, and other support for indigent patients, resulting in marked improvement in adherence to treatment and management among indigent patients seen at NBSCCs
Inadequate disease tracking	Created NBSCC Online Registry for long-term patient tracking
Variable, unstable, or no contingency plans available in many facilities	Developed advisories, guidelines and protocols in times of emergency operations

## Abbreviations

CDC	Centers for Disease Control and Prevention (U.S.)
CH	Congenital hypothyroidism
CHD	Center for Health Development (regional health office)
DILG	Department of the Interior and Local Government
DOH	Department of Health (national health agency, Philippines)
ENBS	Expanded newborn screening
G6PD	Glucose-6-phosphate dehydrogenase
GAL	Galactosemia
LGU	Local Government Unit
MSUD	Maple syrup urine disease
NBS	Newborn bloodspot screening
NBSCC	Newborn screening continuity clinic (long-term follow-up)
NCNBSS	National Comprehensive Newborn Screening System
NIH	National Institutes of Health (Philippines)
NNSGRC	National Newborn Screening and Global Resource Center (U.S.)
NSC	Newborn Screening Center (screening laboratory)
NSF	Newborn Screening Facility (screening specimen collection site)
NSRC	Newborn Screening Reference Center (Philippines)
PEAS	Performance Evaluation and Assessment Scheme (U.S version)
PhilHealth	Philippine Health Insurance Corporation (national health insurance)
PIR	Program implementation review
PKU	Phenylketonuria
PPEAS	Philippine Performance Evaluation and Assessment Scheme
TPMF	Taiwan Preventive Medicine Foundation
